# A Microfluidic and Numerical Analysis of Non-equilibrium Phase Behavior of Gas Condensates

**DOI:** 10.1038/s41598-024-59972-x

**Published:** 2024-04-25

**Authors:** Desmond Batsa Dorhjie, Dmitrii Pereponov, Timur Aminev, Azat Gimazov, Denis Khamidullin, Dmitry Kuporosov, Michael Tarkhov, Alexander Rykov, Ivan Filippov, Elena Mukhina, Evgeny Shilov, Pavel Grishin, Alexey Cheremisin

**Affiliations:** 1 Skolkovo Institute of Science and Technology, CPSE, Moscow, Russia; 2 LABADVANCE LLC, Moscow, Russia; 3 Bashkir State University, Ufa, Russia; 4 Ufa State Petroleum Technological University, Ufa, Russia; 5 Tyumen State University, Tyumen, Russia; 6 Institute of Nanotechnology of Microelectronics of the Russian Academy of Sciences, Moscow, Russia

**Keywords:** Non-equilibrium thermodynamics, Phase separations, Aerosol, Condensate saturation, Relaxation time, Crude oil, Natural gas

## Abstract

Conventional assumptions about multiphase flow in gas condensate reservoirs often do not correlate with field production. This discrepancy stems from the various mechanisms influencing the multiphase process, which are inadequately represented in numerical models. One of the least understood mechanisms is the influence of the non-equilibrium thermodynamics on the flow in the wellbore region, where the reservoir pressure is below the dew point pressure. To address this problem, experimental and mathematical analyses were conducted using a microfluidic device designed to replicate the flow dynamics in a gas condensate system. The experimental results showed an 11% deviation from the initial pressure of condensate saturation when compared with the conventional assumption of local equilibrium in numerical models. Similarly, there is a 14% deviation between the experimental and simulated volumes of the condensate. These findings underscore the inadequacy of existing models to accurately predict the saturation profile of the condensate phase. A mathematical model based on a relaxation parameter was applied to account for non-equilibrium phase separation and the fog state of the aerosol as observed in the microfluidic experiment. Incorporating a relaxation parameter ($\tau$) enhanced the accuracy of the prediction of the initial pressure of the condensate saturation and an improvement in the prediction of the condensate volumes from 76% to 97.2%. Consequently, it provides a valuable framework and insight on the non-equilibrium phase behavior of gas condensate systems under constant flow regimes.

## Introduction

In the next few decades, natural gas is poised to become one of the leading energy sources, driven by the intersection of two key global factors: the surge in energy demand attributed to population growth and the imperative energy transition prompted by climate change^1,2^. Notably, natural gas has a comparatively lower carbon footprint in contrast to coal and oil^3^. This environmental advantage positions natural gas as a pivotal player in meeting the escalating global energy demand^1^. At present, approximately a quarter of the world’s total energy consumption is attributed to natural gas^1^. Furthermore, the global demand for gas is anticipated to rise with an average annual growth rate of 1.6% from 2022 to 2026^1^.

Microdevices and micromodels play pivotal roles across various industries and technological domains. Traditionally, micromodels find extensive application in fields such as medicine, biotechnology, and the chemical industry. Nevertheless, there has been a notable surge in their utilization in diverse sectors, including the oil and gas industries, in recent years. The evolution in the deployment of micromodels is particularly evident in experimental processes aimed at measuring the physical and chemical characteristics of crude oil or natural gas. These measurements include pressure-volume-temperature (PVT)^4,5^, minimum miscibility pressure (MMP)^6^, interfacial tension (IFT)^7–10^ and flow mechanisms^11^. These advancements have yielded benefits, notably in terms of cost reduction, and the reduction of material and time requirements, compared to conventional methodologies. Furthermore, these advancements have led to improvements in measurement precision. For instance, researchers have developed a novel micromodel and experimental method, specifically designed to accurately measure the dew point pressure of gas condensate systems^12^.

**Figure 1:**
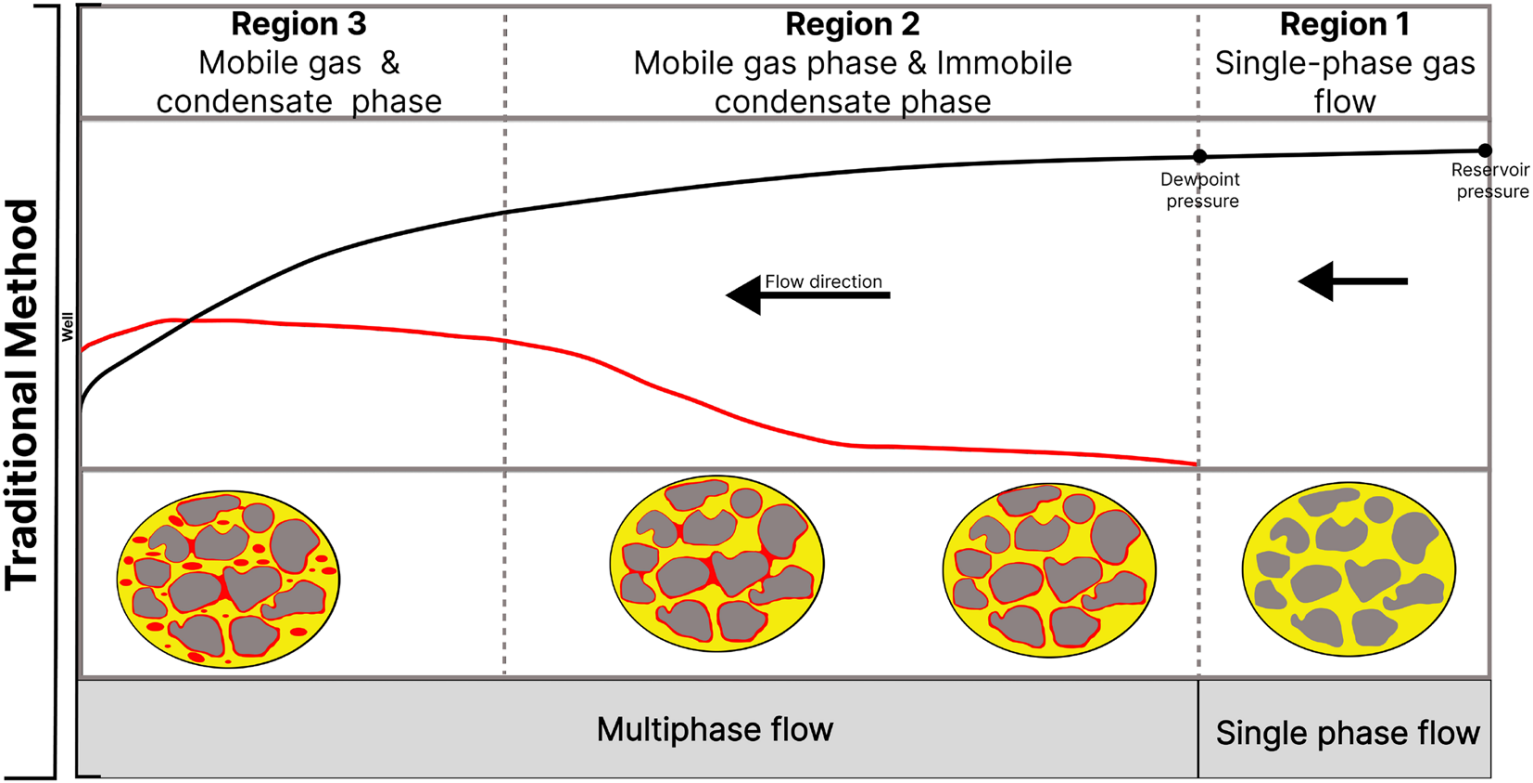
Conventional concept of fluid saturation profile in gas condensate reservoirs.

Gas condensate reservoirs are one of the types of natural gas reservoirs^13^. Gas condensate reservoirs are faced with a peculiar problem known as the condensate bank. The condensate bank develops as a result of the accumulation of immobile condensate in the wellbore region, where the reservoir pressure is below the dew point pressure. The immobile condensate phase blocks pores and reduces the well productivity index of the reservoir up to about 50%^14–17^. In the wellbore region, the saturation profiles of the gas and the condensate phases are divided into three main zones^18^. The first zone is the single-phase flow zone at a distance farthest from the well, where the reservoir pressure is above the dew point pressure. The second zone is the initial saturation zone, where the reservoir pressure is just below the dew point pressure. This zone is characterized by a mobile gas phase and an immobile condensate phase. The third zone is the saturated zone, where the condensate phase saturation is above the critical condensate saturation and hence mobile^13^ (Fig. 1). At the pore scale, the process of condensate formation is as follows; at the infinite boundary of the reservoir, where the pressure exceeds the dew point pressure, there is a uniform flow of a single gas phase. In regions closer to the wellbore where the reservoir pressure is below the dew point pressure, condensate forms as drops and thin film on the rock surface, occupying smaller pores and sharp corners until a local hydraulic conductance of the film is established^19,20^.

Many studies have reported that, in order to accurately model the multiphase flow process in gas condensate reservoirs, the relative permeability has to be expressed as a function of fluid saturation, flow velocity, interfacial tension force (IFT), and wettability^21–23^. Previous experimental investigations presented the negative inertia effect of velocity on the relative permeability and the positive coupling effect of the condensate phase on the relative permeability^23^. Regardless of the improvement in the correlations of the relative permeability, other studies have reported that, due to the volatile nature of gas condensates, the process of filtration is controlled by non-equilibrium thermodynamic mechanisms. These mechanisms include, diffusion^24^, phase change (separation)^20,25,26^, and capillary condensation^27^.

The study of the non-equilibrium thermodynamics of gas condensate revealed a noticeable reduction in effective diffusion with increasing saturation^24^. Furthermore, a clear inverse correlation emerged between pore sizes of the medium and mass transfer coefficients, indicating that smaller pores were associated with higher coefficients between the phases. The conclusion drawn was that the magnitude of mass transfer coefficients is significant, even under low gas flow rates. The study emphasized that the assumptions of local equilibrium in each grid block in the modeling of gas condensate systems could deviate from the saturation profiles in the reservoirs. Experimental studies suggest that in most practical scenarios, non-equilibrium phase behavior can be viewed as a locally equilibrium process^26^. In other cases, local equilibrium is assumed to reduce complexity and computational costs. For gas condensate reservoirs, studies have shown that the assumption of local equilibrium could lead to wrong evaluation of the production and saturation volumes^24,26^. Hence, non-equilibrium processes such as diffusion and dispersion pressure have to be incorporated into simulators for the accurate characterization of the filtration process^24^.

An earlier study emphasized that, the phase separation of gas condensate could be non-instantaneous. A unified non-equilibrium phase behavior model for stand-alone and compositional flow simulations was introduced^26^. The model of non-equilibrium phase behavior requires no modifications to the form of compositional flow equations but only corrections to the flash problem formulation. The model was used to predict actual field production data of heavy component production by the variation of the relaxation time as an adaptation parameter. Numerical analysis of the effect of phase change and non-Darcy flow on gas condensate production presented four different zones of dynamic behavior of the flow^28^, which is an improvement on the traditional three-zone flow conceptual model. These four zones of flow were later confirmed in other studies experimentally^25,29^. Experiments showed that at the dew point pressure, the formation of a condensate is not instantaneous and needs a relaxation time for the condensate phase to fully separate from the gas phases. The experimental studies indicated that there is another flow region between the first and second flow regions of the traditional flow conceptual model. Some studies referred to this zone as the pseudo-single-phase zone with sub-critical liquid nuclei^29^. Further experimental studies have shown that, depending on the depletion rate, which is a function of absolute pressure and pressure gradient (velocity), the volume of condensate produced could vary due to the fog state of the aerosol at higher pressure just below the dew point^25^. This study also confirmed the effect of aerosols on the recovery process, and it was referred to as the fog state zone^25^. Additional investigation showed that gas injection increases the condensate phase recovery when the reservoir pressure is within the pressure of the fog state region^30^.

While most of the studies have shown a deviation of the filtration in gas condensate reservoirs from the traditional assumption of local equilibrium and also the effect of the aerosol state on the condensate production, to the best of our knowledge, none of the previous studies have presented how the non-equilibrium phase separation and the aerosol nature of the condensate phase affect the saturation profile of the condensate. In this study we present a microfluidic experiments to give a visual characterization of the condensate saturation profile under non-equilibrium phase separation and the dynamics of the aerosol (fog) state of the condensate phase. In addition, we provide a mathematical analysis of the flow process and presented a correction parameter to match the condensate saturation profile as observed in the experiment. In this study the word non-equilibrium and non-instantaneous are used interchangeably. Also, the words fog and aerosol are used interchangeably.

## Materials

### Fluid composition

**Figure 2:**
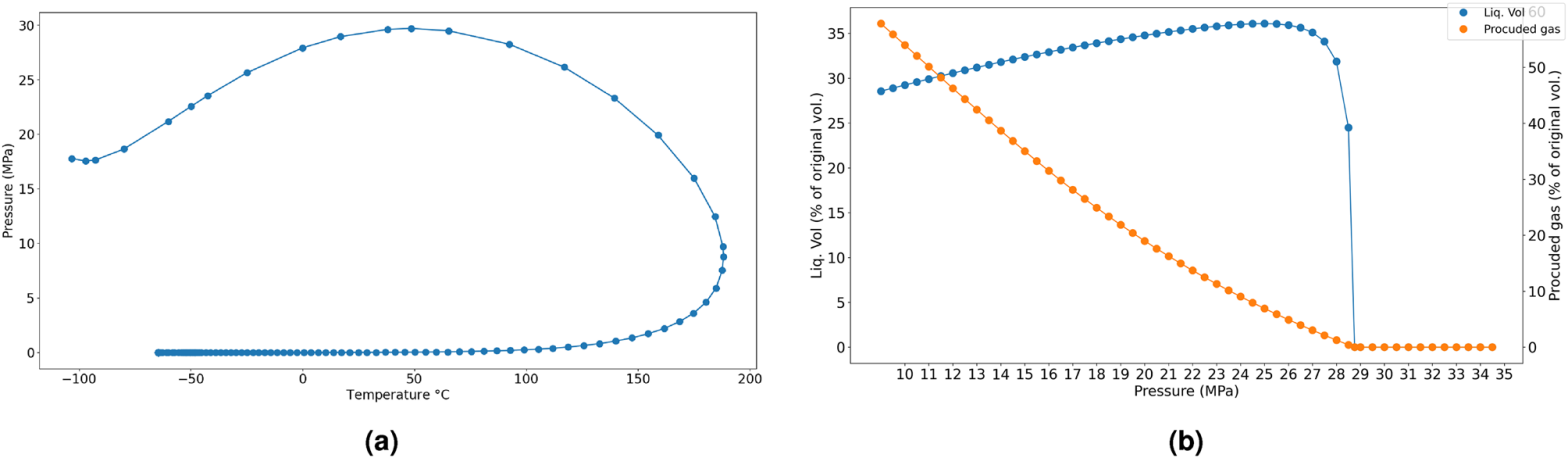
(**a**) Phase envelope of condensate mixture (**b**) Constant volume depletion of the gas condensate mixture.

A three component hydrocarbon mixture was used in this study. The mixture consisted of methane, butane, and decane at 0.8325, 0.1125 and 0.055 mole fractions, respectively. The phase behavior of the gas condensate mixture was obtained from numerical simulation in a commercial simulator based on Peng Robinson equation of state (Fig. 2a). The dew point pressure is 29.0 MPa. The maximum liquid drop out from the CVD simulation is 36.08% at a pressure of 25 MPa as shown in Fig. 2b.

### Micromodel design

**Figure 3:**
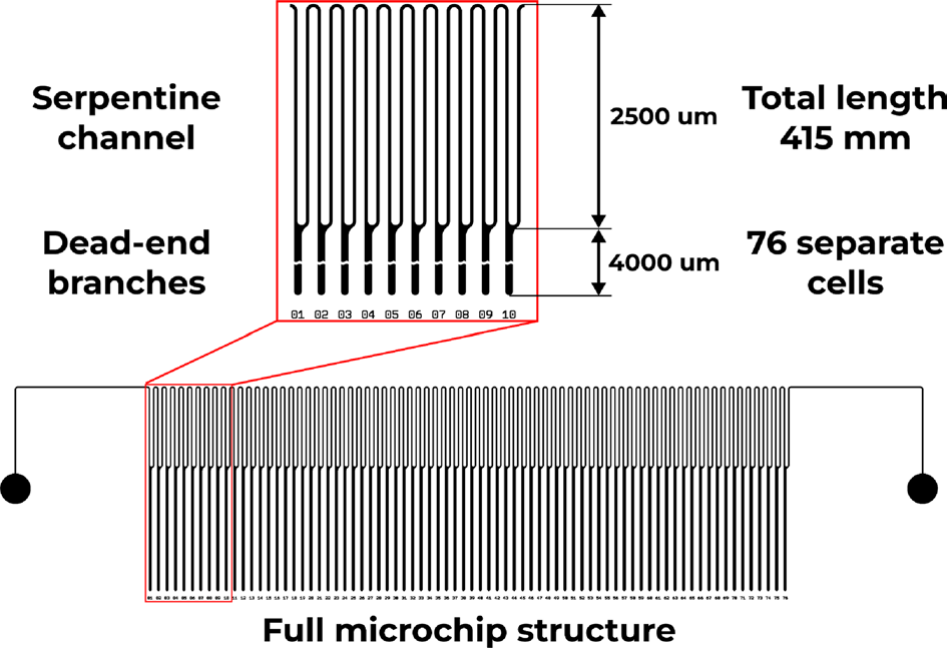
Schematic representation of the micro model.

A silicon-glass micromodel was employed for the experiment. The micromodel was designed in a comb-shaped channel arrangement. The *shaft* is made up of a serpentine channel (Fig. 3). This configuration enabled the shaft to function as a pathway for gas phase flow from the inlet to the outlet. The dead-end branches of the comb facilitated the accumulation of condensate phase when the model was vertically oriented. The entire length of the micromodel is about 415 mm with a total of 76 separate *dead-end branches*. The channels on the micromodel have a depth of 50 $\mu$m and a width of 40 $\mu$m. The microfluidic chip was designed with a single inlet and a single outlet. The *serpentine channel* is about 2500 $\mu$m long in one direction. The schematic representation of the micromodel is presented in Fig. 3. A detailed process of manufacturing the micromodel can be found elsewhere^6^.

### Experimental setup

**Figure 4:**
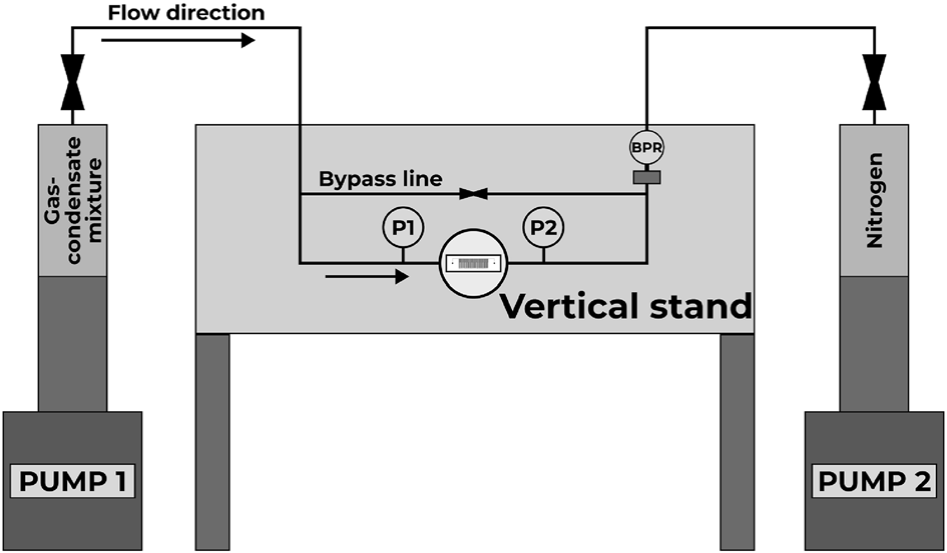
Experimental setup.

The experimental setup consisted of several elements, including a microfluidic chip, a microfluidic holder designed for high-pressure and high-temperature conditions, a pump capable of handling high pressures, valves, and high-speed camera for visual monitoring. To ensure exact fluid control, a laboratory piston pumps capable of high-pressure and high-temperature conditions (LNP, Geologika, Novosibirsk, Russia), with a minimum flow rate set at 0.0001 mL/min, was utilized. A costumed microfluidic holder was used to ensure pressure-tight injection of fluid into the micromodel^31^. To achieve a leakage-free seal between the microfluidic holder and the microfluidic chip, an oil-resistant hard Buna-N o-ring (1247N132, McMaster-CARR, Robbinsville, NJ, USA) was employed. The hydraulic system components were vacuumed using a dual-stage vacuum pump (V-i240SV, VALUE, Wenling China). Outlet pressure was regulated by a backpressure regulator (ZF Zero Flow BPR, Equilibar, Fletcher, NC, USA), controlled with N$_2$ gas. The gas condensate mixture injection was facilitated through a high-pressure transfer vessel. Figure 4 illustrates the experimental setup in a vertical orientation.

## Methodology

### Experimental sequence

The experiment was conducted at room temperature of 20 °C. The sequence of the experiment is as follows, first the micromodel was set in-place to the micromodel holder and oriented in a vertical position. The system was vacuumed for thirty minutes. Then, a leakage check was conducted by injecting helium at a pressure 25% higher than the initial pressure of the experiment. Next, the pressure was lowered, and the system was vacuumed again. Methane was further injected into the micromodel to avoid the loss of the liquid phase of the gas condensate sample. After the process of methane injection for about 12 PV, the gas condensate mixture was then injected into the model at a pressure above the dew point pressure (29.3 MPa). Once a steady state was reached the pressure at the outlet of the model was decreased from 29.3 MPa to about 23.4 MPa. At regions of the model where the pressure was below the dew point pressure, the condensate began to form and was collected in the cells perpendicular to the flow direction (“dead-end branches”). Continuous injection of the gas condensate mixture, at the inlet, was maintained throughout the experiment to keep the pressure at the inlet above the dew point pressure. Similarly the pressure at the outlet was kept at 23.4 MPa until the cells were fully saturated with the condensate. The evolution of the condensation and saturation profile of the condensate phase was recorded with a high-resolution camera.

## Results and discussion

### Experimental Results

**Figure 5:**
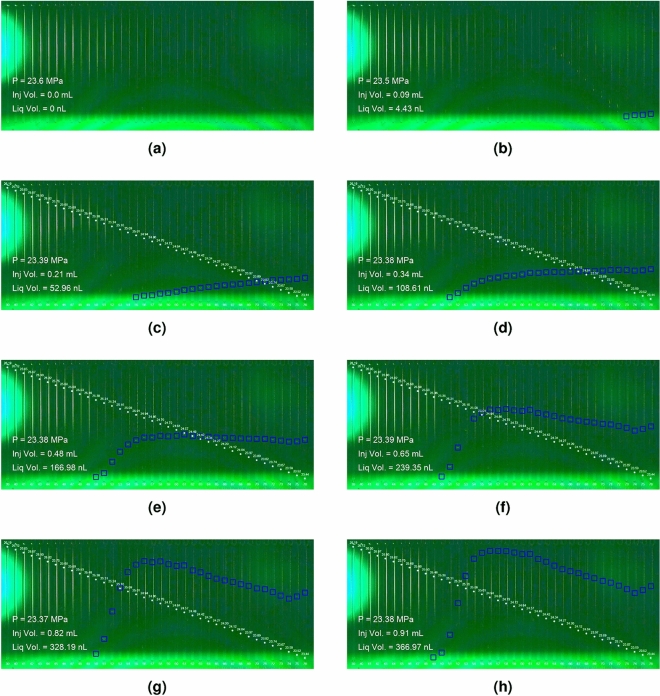
Evolution (**a**–**h**) of condensate saturation in the cells of the micro model.

The process of condensation and the evolution of the saturation profile is presented in Fig. 5. The first accumulation of the condensate was detected at the cell closest to the outlet of the micromodel—the region of lowest pressure in the system (Fig. 5b). The accumulation of high condensate volume at the cell closest to the outlet continued for a limited time (Fig. 5b–d). As the process continued, the condensate volume in the cells at the boundary of the initial condensate saturation began to increase faster than the volume in the cells closest to the outlet (Fig. 5e–h). The evolution of the saturation map of condensate phase agreed with the profile of condensate saturation from analysis of reservoir well testing data^18^. The deviation of the last two cells closest to the outlet from the trend of the saturation profile in the experiment can be attributed to capillary end effects^32^. The flow in the experiment was confirmed to be laminar flow with a Reynolds number of 52.. Additional video of the evolution of the saturation profile and method of image and video processing can be found in the Supplementary materials 1 and 2, respectively.

**Figure 6:**
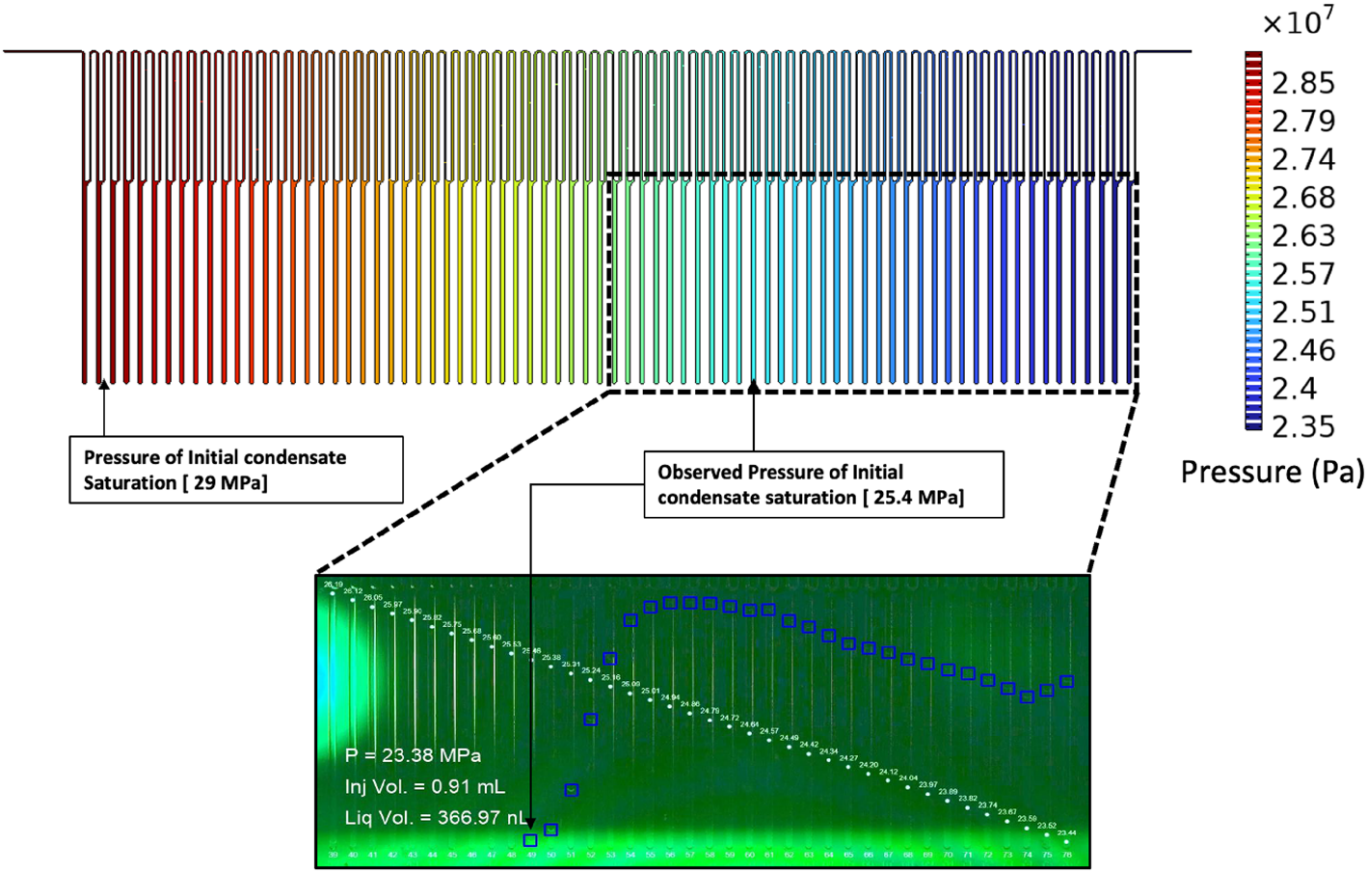
Representation of pressure distribution in the micro model.

For further analysis and interpretation, a computational fluid dynamics (CFD) simulation of the flow in the micro channel was computed to identify the pressure distribution in the micromodel. A 2D representation of the micromodel was used for computing the pressure distribution. The physical properties of the gas phase (density and viscosity) were computed by cubic equation of state. The inlet and outlet pressure used in the experiment were used as the boundary conditions for the simulation. The result showed a linear pressure distribution in the micromodel (Fig. 6). Comparison of the pressure profile and condensate saturation profile showed that, the pressure at which condensate began to form, as observed in the experiment, deviated from the dew point pressure originally computed by the cubic equation of state for the gas condensate mixture (Fig. 2b). In the experiment, the cell with the initial condensate saturation in the direction of the flow had a pressure of 25.46 MPa (cell 49). Meanwhile, the expected value from the cubic equation of state was approximately 29 MPa (cell 3) as shown in Fig. 6. This is about 11% of shift in the saturation map of the condensate towards the outlet. These findings validate the hypothesis from previous studies which suggested that, while the condensate formation is instantaneous, the mechanism of phase separation of the condensate from the gas is not instantaneous^25,29^. Hence, the condensate at the initial stage flows in the form of aerosols with the gas phase (fog state) in the direction of the flow and is latter trapped in the dead-end branches when complete phase separation was achieved.

### Material balance model

The deviation of the initial saturation pressure of the condensate phase as observed in the experiment from the numerical equilibrium equation of state shows that, the current mathematical models that rely on equilibrium equation of state in the computation of condensate saturation profile and production volumes for gas condensate reservoirs could lead to an overestimation of the size of the condensate bank and an underestimation of the early stage production volumes. In this section, a material balance model to account for the fog state of the condensate phase is proposed and validated. In the context of flow in porous media, a material balance is a fundamental concept used to describe the behavior of fluids within porous media, such as soils, rocks, or sediments. It is commonly applied in various fields, including hydrogeology, petroleum engineering, and environmental science. The material balance equation for flow in porous media is derived from the principle of conservation of mass. Mathematically, the material balance equation for flow in porous media can be expressed as shown in Eq. (1).

1 $$\begin{aligned} \frac{\partial \left( \phi \cdot S \cdot \rho \right) }{\partial t} + \nabla \cdot \left( \rho \cdot {\textbf {v}}\right) = 0 \end{aligned}$$

Where $\phi$ is the porosity of the porous media (dimensionless), *S* is the fluid saturation (dimensionless), $\rho$ is the density of the fluid (${\text{kg}}/{\text{m}}^3$), *t* is the time (*s*), ${\textbf {v}}$ is the Darcy velocity vector (*m*/*s*). The term $\nabla \cdot \left( \rho \cdot {\textbf {v}}\right)$ represents the divergence of the mass flux vector. The conservation of momentum in porous media flow for a given phase is traditionally expressed with the Darcy law^33^. Considering a 1 dimensional rigid isothermal medium with an incompressible phase ($\alpha$) flowing at a steady state, Eq. (1) can be written as Eq. (2).

2 $$\begin{aligned} \frac{ \partial (\phi S_{\alpha })}{\partial t} + \frac{\partial \upsilon _{\alpha }}{\partial x} = 0 \end{aligned}$$

To ensure a good comparison of the micromodel experiment with the macro scale formulation of the mass and momentum conservation laws, some assumptions were made. First, the filtration of the gas between the inlet and the outlet is considered to be one-dimensional. Also, the porosity of the 1D model was regarded as 1 since the channels of the micromodels have a total void. The phases ($S_\alpha$) were solved by the so-called “flash calculation” at each time step, utilizing the Peng-Robinson cubic equation of state. Since the micromodel was designed to collect the condensate (liquid) in the dead-end branches, the condensate was regarded as immobile in the flow direction. Hence the gas phase relative permeability is regarded as equal to 1 ($k_{rg} = 1$) and the condensate phase relative permeability is regarded as equal to zero ($k_{ro} = 0$). Furthermore, to ensure comparison of the mathematical model with the experimental results, the material balance equation is presented in a non-dimensionless form. The non-dimensional form allows the validation of the model with existing macro scale hydrodynamic simulators and also comparisons with the millimeter scale experimental results presented. The dimensionless spatial coordinate ($x^*$) in the x-direction is given as $x^* = \frac{x}{L}$, the dimensionless time is given by $t^* = \frac{t}{T}$, the dimensionless velocity ($v^*$) is given as $v^* = \frac{x^*}{t^*}$. Where *L* and *T* are the characteristic length and time scales from the experiment. Based on the assumptions given, at a constant flow velocity, the equation for the conservation of mass (Equation (1)) can be written in the simplified dimensionless form as Eq. (3) for the gas phase and as Eq. (4) for the condensate phase. Since in the experiment, a gas condensate mixture was continuously injected into the model via the inlet, an additional source term (*q*) appears on the right side of Eq. (3).

3 $$\begin{aligned} \frac{d S_g}{d t^*} + v^*_g= & {} q \end{aligned}$$

4 $$\begin{aligned} \frac{d S_o}{ d t^*}= & {} 0 \end{aligned}$$

#### Model validation

**Figure 7:**
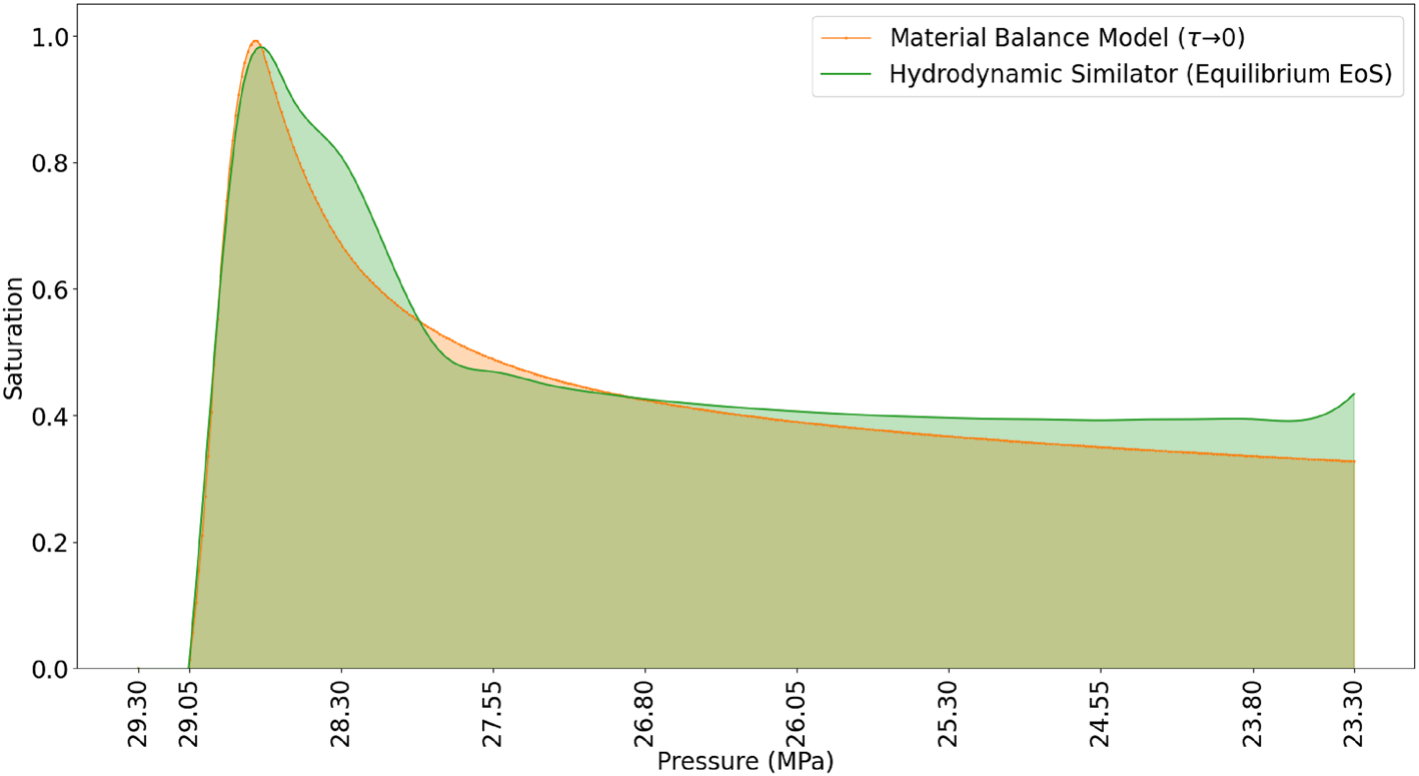
Comparison of the material balance model and hydrodynamic simulator: simulated under equilibrium conditions. (Flow direction is from left to right).

The non-dimensional material balance model was solved for using the linear pressure distribution in the model between the inlet and the outlet as presented in the experiment. The material balance model was validated by comparison with the results from a commercial compositional hydrodynamic simulator. The hydrodynamic model was developed based on the same specification of the 1D material balance model. The models were simulated until the saturation of the condensate phase in a cell reaches 100%. Figure 7 presents the result of the material balance model and that of the hydrodynamic simulator. The pressure of initial saturation is identical in both simulations (29 MPa). Furthermore, the volume of condensate saturation from and the saturation profile along the flow direction are also identical: 41.73% and 44.26% for the material balance model and the hydrodynamic simulator respectively (an agreement of 97.5%). This shows that the presented simplified material balance model can be used to estimate the dynamics of the condensate saturation on different scales: macro and millimeter scales.

**Figure 8:**
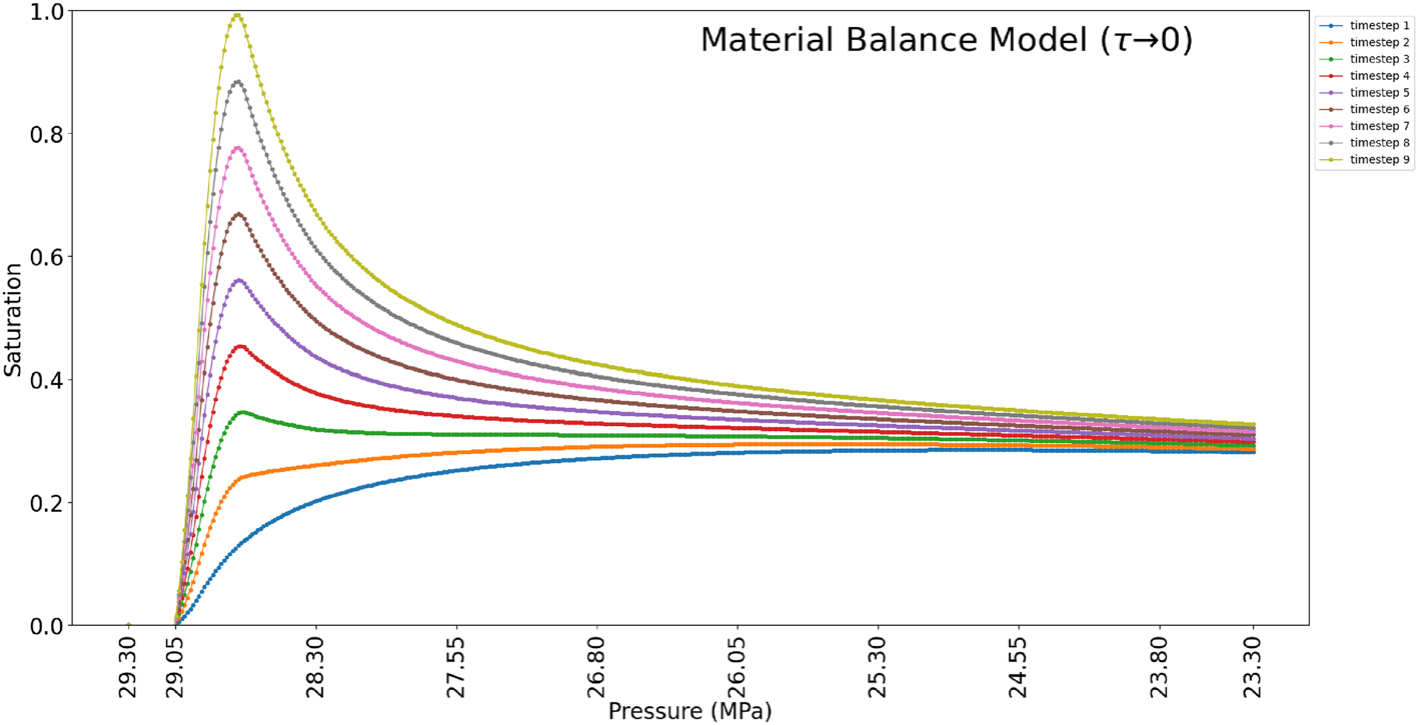
Evolution of condensate saturation profile under equilibrium condition (instantaneous condensation and phase separation).

Furthermore, the presented material balance model is capable of predicting the evolution of the saturation profile of the condensate phase as observed in the experiment (Fig. 8). Each time step signifies the saturation of the immobile condensate phase in the dead-end cells along the direction of the flow. From the results (Fig. 8), it can be seen that at the initial stages of the saturation, the condensate saturation is higher in the cell closest to the outlet: similar to the experiment. As the flow continues, the saturation profile changes and the zone towards the inlet increases. The condensate saturation towards the inlet increases at a faster rate as compared to the condensate at the outlet. This is due to the fact that, at the inlet, the initial composition of the gas condensate mixture is continuously injected into the micromodel (this is to simulate the infinite boundary of the reservoirs with single phase gas flows.). The in-flow gas condensate mixture has a higher mole ratio of heavier components as compared to the other zones of the micromodel and hence higher volumes of condensate saturation. Towards the outlet, the gas phase has a lower mole ratio of the heavier components (lean gas) and, hence, little increase in condensate saturation.

#### Non-equilibrium phase separation of condensate from gas phase

**Figure 9:**
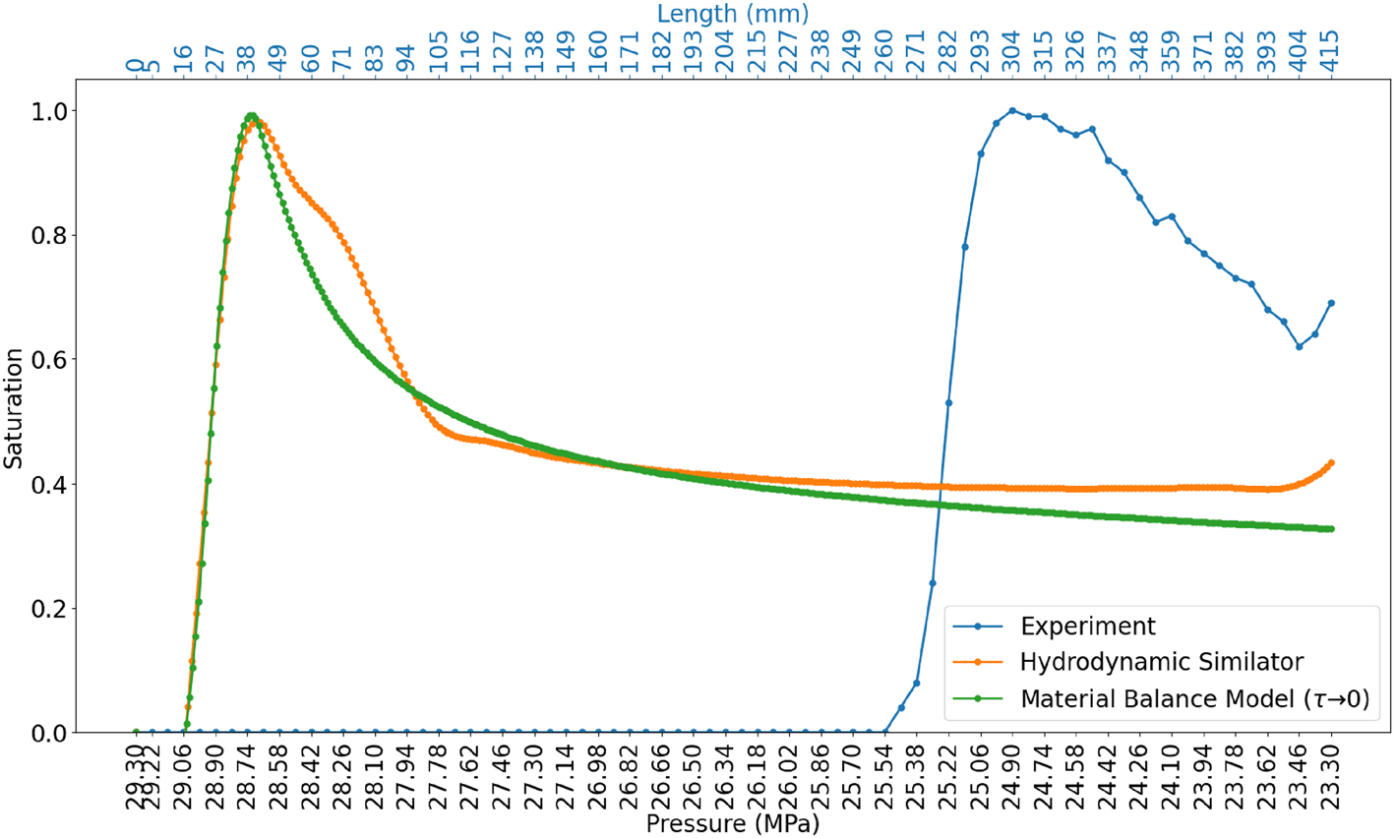
Comparison of the experimental results, the material balance model (simulated under equilibrium conditions) and hydrodynamic simulator (simulated under equilibrium conditions). The secondary x-axis is the length scale of the micromodel.

The simulated results of the material balance model and the hydrodynamics simulator were compared with the experimental results (Fig. 9). The results show that the pressure of initial condensate saturation deviates from the result of the material balance model and the hydrodynamic simulator (Fig. 9). The saturation profile of the condensate shifts more towards the outlet in the direction of the flow. It should be considered that, the deviation is mainly due to the fact that, in the hydrodynamic modeling, the thermodynamic equation of state is based on the assumption of a local equilibrium at each time step. The condensation of the liquid phase is regarded instantaneous. In contrast to the experiment, the condensate phase is initially carried in the gas phase as aerosols (fog state) along the flow direction until a complete phase change is established. At the point of complete phase change, the condensate phase is captured in the dead-end branches and remains immobile in the direction of the flow. This signifies that the assumption of local equilibrium and instantaneous phase change for the modeling of two-phase flow can incorrectly estimate the saturation profile of the condensate phase. Due to the shift in the initial pressure of saturation, the volume of condensate formed is also altered. The material balance model with the assumptions of local equilibrium predicted a 41.73% saturation of the condensate phase, while only 27.42% saturation was observed in the experiment.

To account for limitation of the presented material balance model and also existing hydrodynamic models, a concept of relaxation parameter ($\tau$) is applied to account for the non-instantaneous phase separation. To incorporate the relaxation parameter into the material balance model, additional steps are required when solving for the fluid saturation ($S_\alpha$). First, the condensate saturation ($S_o^{eos}$) and gas saturation ($S_g$) are solved for by the “Flash calculations”. From the hypothesis of non-instantaneous phase separation, the $S_o^{eos}$ solved for by the “Flash calculation” has two components: the mobile condensate phase in the form of aerosol - fog state ($S^{fog}_{o}$) and the immobile condensate phase that is trapped in the dead-end branches - effective condensate ($S^{eff}_{o}$). The effective condensate saturation (immobile condensate) is solved for by Eq. (5). Where *C* is the dimensionless coefficient of hydraulic resistance force acting against the flow. The hydraulic resistance to the flow can be described in terms of the petrophysical characteristics of the porous media (porosity, permeability). However, in this study, the resistance to flow is not considered, hence $C=1$.

5 $$\begin{aligned} S^{eff}_{o} = \left( S^{eos}_{o} + S^{fog}_{o} \right) - e^{\frac{-1}{\tau } \cdot C} \end{aligned}$$

**Figure 10:**
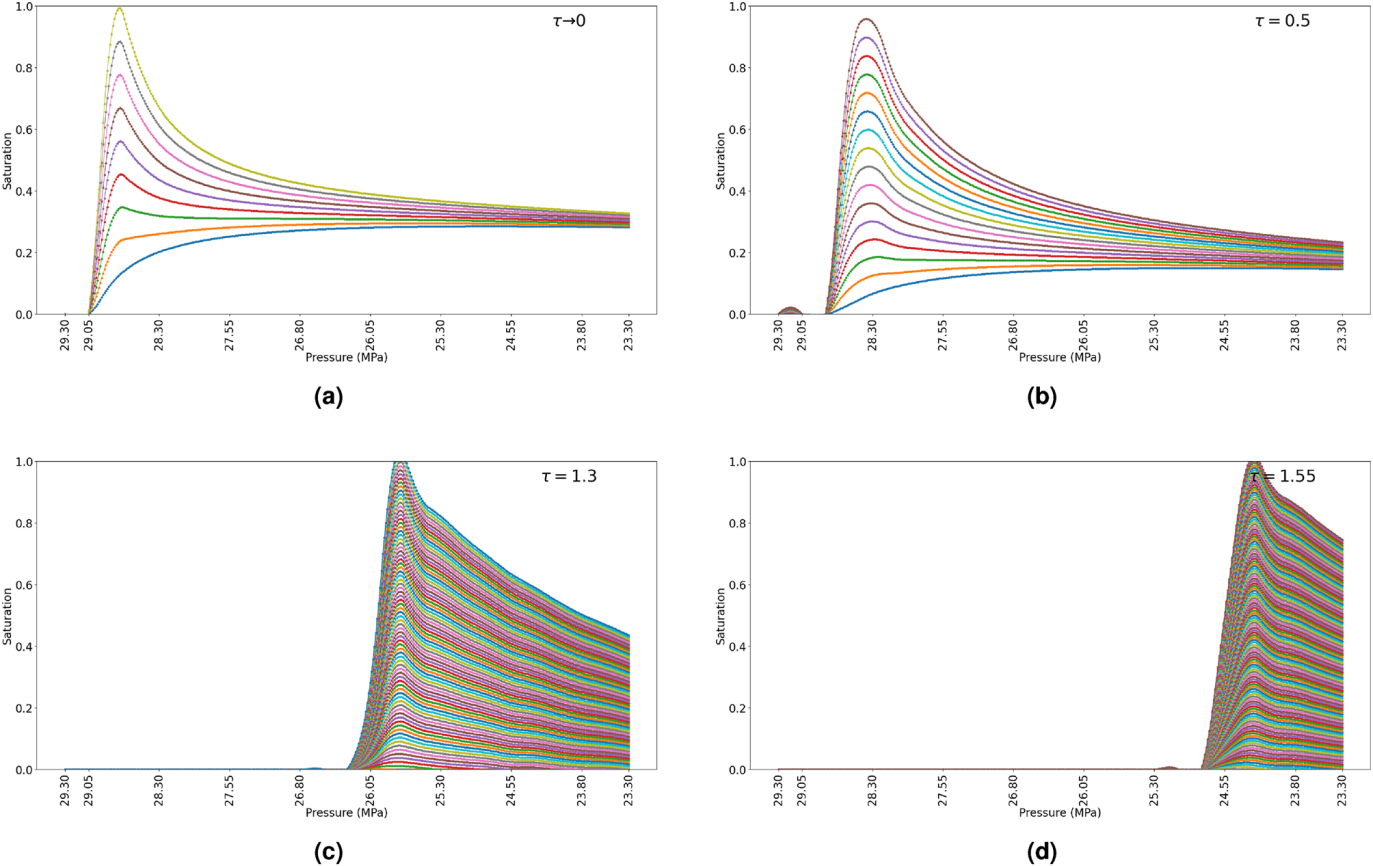
Saturation profile of condensate (**a**) Evolution of condensate phase when $\tau \rightarrow 0$ (**b**) Evolution of condensate phase when $\tau = 0.5$ (**c**) Evolution of condensate phase when $\tau = 1.3$ (**d**) Evolution of condensate phase when $\tau = 1.55$.

The results show that when $\tau$ approaches 0, the curve is equal to the simulations based on the assumption of instantaneous phase separation (thermodynamic equilibrium). When $\tau>> 0$ the results show a shift in the saturation profile (Fig. 10). The pressure at which the saturation profile from the inlet towards the outlet starts at $\approx 29.06, 28.8, 26.3$ and 24.7 MPa when $\tau \approx 0$, $\tau = 0.5$, $\tau = 1.3$ and $\tau = 1.55$ respectively. In addition, the number of dimensionless time steps for the cells to be completely filled is increased. The number of time-steps for the saturation to reach 100% is 10, 17, 92, 147 when $\tau \approx 0$, $\tau = 0.5$, $\tau = 1.3$ and $\tau = 1.55$ respectively.

**Figure 11:**
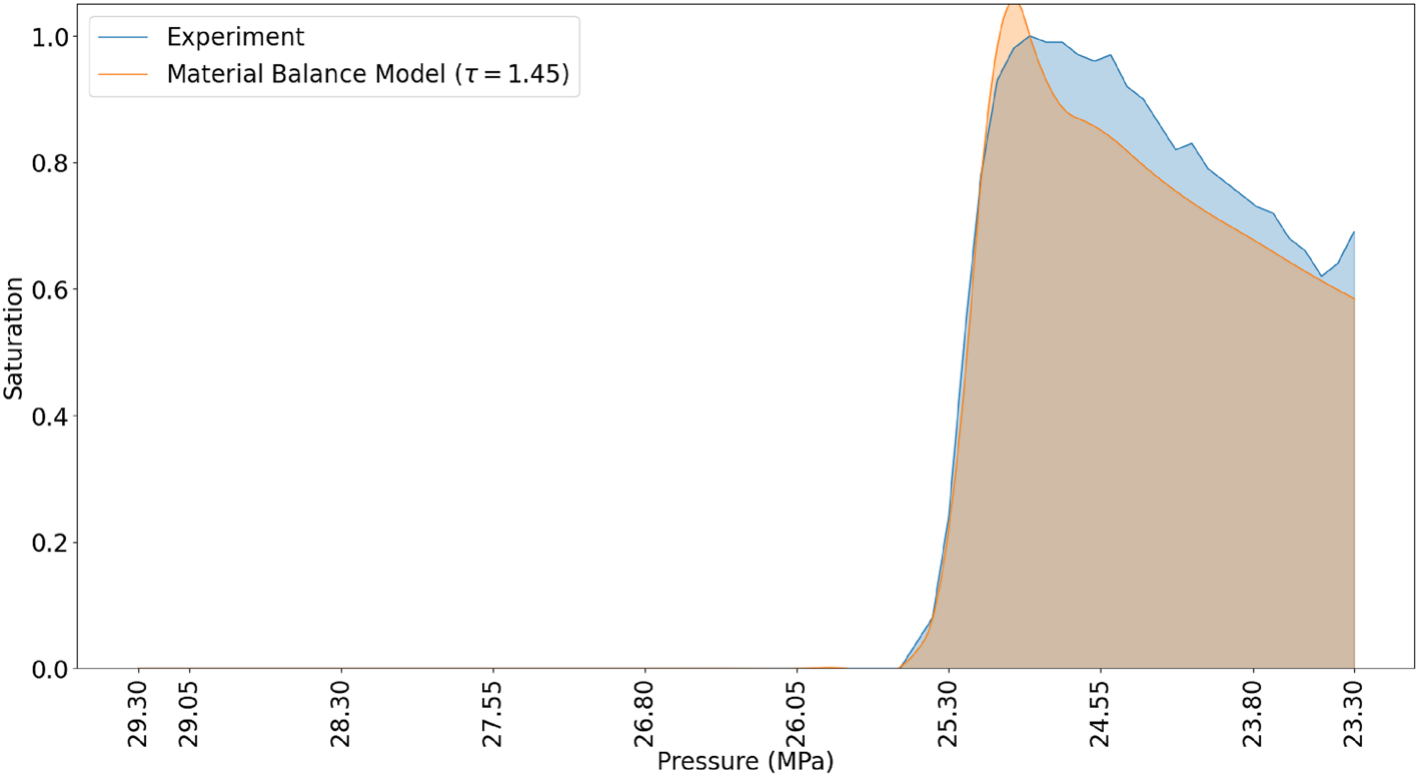
Comparison of result from material balance model and experiment when taking into account non-instantaneous phase separation.

The material balance model was matched with the experimental results at $\tau = 1.45$ (Fig. 11). At $\tau = 1.45$ the pressure of initial condensate saturation corresponds exactly with the the observations from the experiment. Similarly, a 97.2 % agreement in the volume of accumulated condensate in the dead-end branches was observed. This method is a 21% improvement on the simulations where the fog state was not accounted for.

**Figure 12:**
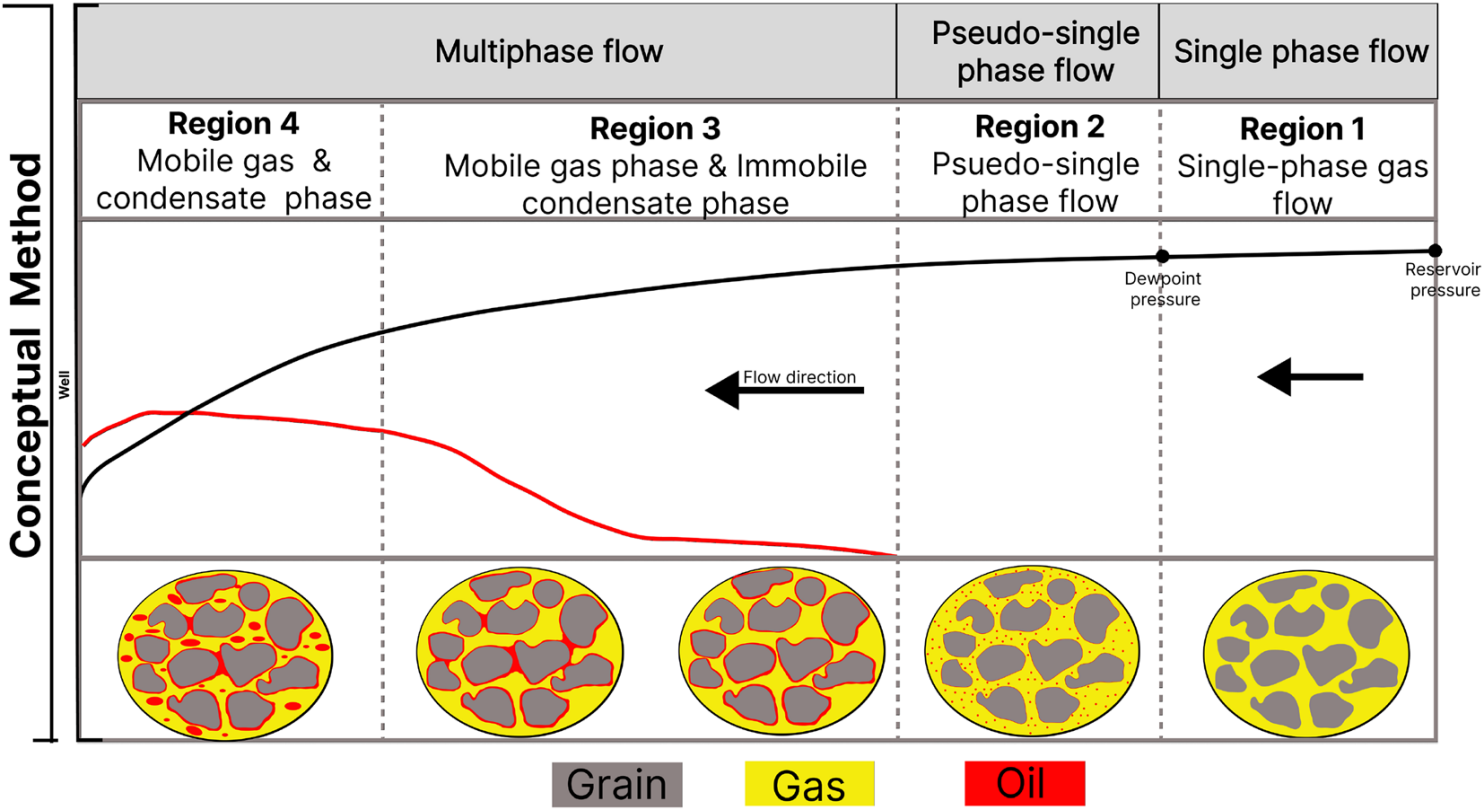
Conceptual model for multiphase flow modeling in the wellbore region of gas condensate reservoirs.

The results presented in this study have demonstrated that the concept of aerosol state and the dynamics of phase separation of the condensate can lead to a shift in the saturation profile from the equilibrium assumptions. These findings show that an additional zone exists between the zone of single phase flow and the zone of immobile condensate flow (Fig. 12).

In the wellbore region, where the pressure is below the dew point, the condensate first forms as nano aerosols and is mobilized as a dispersed phase with the gas towards the wellbore. The mobility of the aerosols is high and could move towards the wellbore depending on the flow velocity and the hydraulic resistance to the flow. The incorporation of the correction factor ($\tau$) provides a relaxation parameter that accounts for the non-equilibrium phase separation and improves the prediction of the evolution of the condensate saturation profile. The accurate estimation of the saturation profile will help in planning the optimal techniques, including methods of enhanced oil recovery, for optimal condensate recovery. While the microfluidic device used in this study does not completely represent the complexity of a porous medium, other experiments utilizing core samples (geological porous media) have demonstrated the occurrence of nano-aerosol and fog state of the condensate and the potential benefit, it could play in the recovery of the condensate^25,29^. Nevertheless, further studies are still required to express the effect of an actual porous medium and the flow velocity on the relaxation parameter and the non-instantaneous phase separation which was presented. In addition, further research is required to upscale the presented mathematical model from a dimensionless form to the reservoir scale, where there is a need to account for the fog state of the condensate phase in hydrodynamic simulators.

## Conclusion

This work presented a microfluidic experiment and mathematical study of the fog state of gas condensate in the wellbore region when the reservoir pressure is below the dew point pressure. The microfluidic experiment provided a visualization of the saturation profile of the condensate, which improves the understanding of non-equilibrium thermodynamics arising from the non-instantaneous phase separation of the condensate from the gas phase under constant flow regime. The condensate, when formed in the region where the pressure is below the dew point, flows as aerosols. This region is termed the fog state zone. These findings confirm an improvement in the conceptual model of gas condensate flow.

Furthermore, a mathematical formulation based on the concept of relaxation parameter ($\tau$) was introduced to account for the fog state of the condensate phase in the flow process. The experiment, when coupled with the proposed mathematical model, enhances the prediction of the relaxation parameter associated with gas condensate saturation in the wellbore region. For the presented experiment, at $\tau = 1.45$, the model accurately predicted the pressure of initial saturation and also improved the prediction of the total volume of condensate saturation from 76 to 97.2%.

### Supplementary Information

Supplementary Information 1.

Supplementary Information 2.

## Data Availability

All data generated or analysed during this study are included in this published article and its supplementary information files.
