# Author Correction: A microfluidic and numerical analysis of non-equilibrium phase behavior of gas condensates

**DOI:** 10.1038/s41598-024-66556-2

**Published:** 2024-07-08

**Authors:** Desmond Batsa Dorhjie, Dmitrii Pereponov, Timur Aminev, Azat Gimazov, Denis Khamidullin, Dmitry Kuporosov, Michael Tarkhov, Alexander Rykov, Ivan Filippov, Elena Mukhina, Evgeny Shilov, Pavel Grishin, Alexey Cheremisin

**Affiliations:** 1 Skolkovo Institute of Science and Technology, CPSE, Moscow, Russia; 2 LABADVANCE LLC, Moscow, Russia; 3 Bashkir State University, Ufa, Russia; 4 Ufa State Petroleum Technological University, Ufa, Russia; 5 Tyumen State University, Tyumen, Russia; 6 Institute of Nanotechnology of Microelectronics of the Russian Academy of Sciences, Moscow, Russia

Correction to: *Scientific Report* 10.1038/s41598-024-59972-x, published online 25 April 2024.

The original version of this Article contained an error in Reference 18, which was incorrectly given as:

Zbiciak, A. & Markiewicz, T. A new extraordinary means of appeal in the polish criminal procedure: The basic principles of a fair trial and a complaint against a cassatory judgment. Access Justice Eastern Europe 6, 1–18 (2023).

The correct reference is listed below:

Gringarten, A. C., Al-Lamki, A., Daungkaew, S., Mott, R. & Whittle, T. M. Well test analysis in gas-condensate reservoirs. In All Days, 00ATCE, 10.2118/62920-ms (SPE, 2000).

The original Article has been corrected.

