# Supplementary material for: A Microfluidic and Numerical Analysis of Non-equilibrium Phase Behavior of Gas Condensates
Source: Sci Rep. 2024 Apr 25;14:9500. doi: 10.1038/s41598-024-59972-x (PMC11045818; doi:10.1038/s41598-024-59972-x)
Supplement: Supplementary file 1 — Supplementary Information 1. [file 41598_2024_59972_MOESM1_ESM.pdf]

A Microfluidic and Numerical Analysis of Non-Equilibrium Phase Behavior of Gas Condensates

Supplementary Material 1

1. Video and Image Processing

The condensate phase was detected based on the meniscus created between the condensate and gas phase and enhanced with the help of video processing. Video processing of experimental video for studying the kinetics of gas condensate sedimentation was implemented in the Python programming language (version 3.10.1). Detection and tracking of the interface boundary between gas and condensate in the gas-condensate mixture were performed using functions from the OpenCV library (version 4.7.0) and involved several stages. Image preprocessing methods such as histogram equalization or Gaussian filtering were applied to enhance image contrast and sharpness, reduce noise, and address other artifacts that may affect subsequent analysis. A crucial subsequent step involved converting the image to greyscale format, which is necessary for further processing. The algorithm's operation principle is illustrated in Supplementary Figure 1. Next, a suitable template image of the phase boundary was manually selected by cropping a small portion of a video frame. To accurately track the movement of the boundary over

time, a template matching algorithm is applied to the entire video using the aforementioned steps. Consequently, the phase boundary is detected on each experimental frame in each cell for condensate accumulation. The volume of the precipitated condensate is calculated based on known parameters of the channel's width and etching depth, as well as the determined height of condensate filling in the cell. An example frame illustrating this is shown in Supplementary Figure 1d. Additionally, the volume of the pumped gas-condensate mixture through the serpentine channel and the pressure at the outlet of the microfluidic chip are also displayed on the frame. Furthermore, a mask indicating the pressure in each condensate accumulation cell is overlaid on the frame. The mask was created assuming that the change in pressure occurs linearly along the length of the microchip.

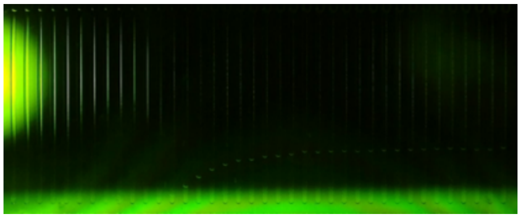

(a)

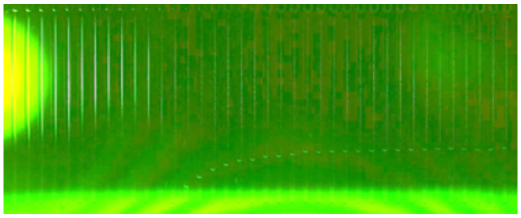

(b)

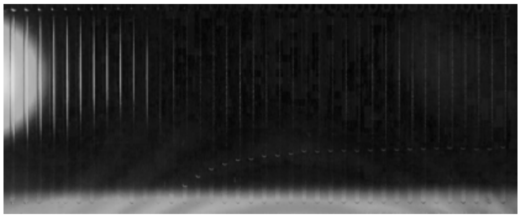

(c)

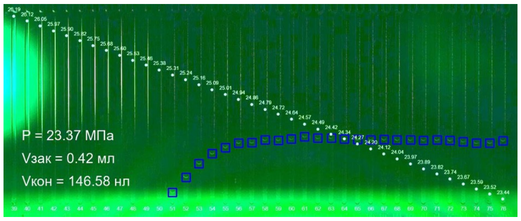

(d)

Supplementary Figure 1: Video processing (a) Original image (b) Image with increased contrast (c) Gray-scale image (d) Image with interface tracking
